# Combined Airway and Bariatric Surgery for Obesity Hypoventilation Syndrome: Feasibility and Early Outcomes—A Prospective Observational Cohort Study

**DOI:** 10.3390/jcm15145679

**Published:** 2026-07-20

**Authors:** Hsueh-Yu Li, Li-Pang Chuang, Ming-Shao Tsai, Keng-Hao Liu, Ya-Wei Hsiao, Wen-Nuan Cheng, Li-Ang Lee

**Affiliations:** 1 Department of Otorhinolaryngology-Head and Neck Surgery, Linkou Main Branch, Chang Gung Memorial Hospital, Taoyuan 33305, Taiwan; hyli38@cgmh.org.tw (H.-Y.L.); hsiaoyawei@cgmh.org.tw (Y.-W.H.); 2 School of Medicine, College of Medicine, Chang Gung University, Taoyuan 33302, Taiwan; r5243@cgmh.org.tw (L.-P.C.); b87401061@cgmh.org.tw (M.-S.T.); kenghao@cgmh.org.tw (K.-H.L.); 3 Department of Pulmonary and Critical Care Medicine, Linkou Main Branch, Chang Gung Memorial Hospital, Taoyuan 33305, Taiwan; 4 Department of Otorhinolaryngology-Head and Neck Surgery, Chiayi Chang Gung Memorial Hospital, Chiayi 613, Taiwan; 5 Department of Surgery, Linkou Main Branch, Chang Gung Memorial Hospital, Taoyuan 33305, Taiwan; 6 Department of Sports Sciences, University of Taipei, Taipei 111036, Taiwan; kara_cheng@yahoo.com; 7 School of Medicine, College of Life Science and Medicine, National Tsing Hua University, Hsinchu 300044, Taiwan

**Keywords:** combined airway and bariatric surgery, daytime hypercapnia, obesity hypoventilation syndrome, obstructive sleep apnea, sleepiness, transdisciplinary intervention

## Abstract

**Background**: Obesity hypoventilation syndrome (OHS) is a life-threatening condition often underdiagnosed in patients with obstructive sleep apnea (OSA). While bariatric surgery induces weight loss, it does not immediately resolve upper airway obstruction. We investigated the feasibility and clinical predictors of a novel transdisciplinary approach—Combined Airway and Bariatric Surgery (CABS)—in treating OHS. **Methods**: This prospective observational cohort study (December 2022–November 2024) recruited 36 adults with obesity (body mass index [BMI] ≥ 30 kg/m^2^) and OSA (apnea–hypopnea index [AHI] ≥ 5 events/h). OHS was diagnosed by daytime hypercapnia (PaCO_2_ ≥ 45 mmHg) after excluding other causes of hypoventilation. Participants received CABS or positive airway pressure therapy via shared decision-making. Primary outcomes included changes in AHI, BMI, PaCO_2_, and Epworth Sleepiness Scale (ESS). **Results**: Nine of 36 individuals (25%) had OHS. Multivariate logistic regression identified BMI and ESS as significant predictors. Receiver operating characteristic curve analysis showed high OHS prediction with BMI ≥ 35.5 kg/m^2^ (area under the curve [AUC] = 0.85), ESS ≥ 12 (AUC = 0.72), and a novel OHS prediction score > 20 (AUC = 0.94). Among 6 OHS patients who underwent CABS, one-year follow-up showed significant improvements: AHI decreased from 98.1 to 26.8 events/h, BMI from 40.1 to 29.1 kg/m^2^, and PaCO_2_ from 50.2 to 37.9 mmHg (all *p* < 0.05). **Conclusions**: OHS was present in one-quarter of individuals with obesity and OSA in this cohort. CABS was feasible and associated with encouraging improvements in sleep-disordered breathing, ventilatory status, and weight among selected patients. These preliminary findings support further evaluation of this transdisciplinary strategy in larger, comparative studies.

## 1. Introduction

Obesity hypoventilation syndrome (OHS) has emerged as a major respiratory complication in parallel with the worldwide rise in obesity. The condition is defined by the combination of obesity (body mass index [BMI] ≥ 30 kg/m^2^), chronically elevated daytime arterial carbon dioxide levels (PaCO_2_ > 45 mmHg), and sleep-disordered breathing (SDB), once other causes of sustained hypoventilation—such as neuromuscular weakness or structural thoracic abnormalities—have been excluded [1,2]. Recent epidemiologic analyses suggest that OHS is no longer a rare entity; approximately 8–20% of adults with obesity meet diagnostic criteria, and the proportion increases to nearly 25% among individuals with morbid obesity (BMI > 40 kg/m^2^) [3].

OHS and obstructive sleep apnea (OSA) frequently coexist, and contemporary reports indicate that the vast majority of patients with OHS also fulfill diagnostic criteria for OSA, with severe OSA present in a substantial proportion of cases [4]. Compared with patients who have OSA alone, those with OHS typically exhibit more advanced obesity, higher apnea–hypopnea index (AHI), lower minimum peripheral capillary oxygen saturation (SpO_2_), and more prominent daytime symptoms such as hypersomnolence and exertional dyspnea [5,6,7]. Despite the overlap, OHS represents a distinct and more clinically aggressive phenotype. Individuals with OHS experience deeper and more prolonged nocturnal hypoxemia, greater impairment in daytime functioning, and substantially increased healthcare utilization when compared with eucapnic OSA patients [8].

The comorbidity profile of OHS is extensive, severely compounding the danger of the disease. Patients routinely present with advanced cardiometabolic complications, including resistant systemic hypertension, type 2 diabetes mellitus, right-sided heart failure (cor pulmonale), and pulmonary hypertension [2,7]. Consequently, the prognosis for unmanaged OHS is exceptionally poor [9]. Observational data reveal that untreated OHS carries an alarming 24% mortality rate within 1.5 to 2 years, with mortality in intensive care settings reaching 31% at three years [3,7]. Such statistics underscore the critical need for timely, comprehensive, and effective therapeutic interventions.

Positive airway pressure (PAP) therapy remains the standard treatment for OHS, with continuous PAP (CPAP) and non-invasive ventilation used to address obstructive events, nocturnal hypoxemia, and hypercapnia [10]. CPAP is the first-line treatment for stable ambulatory patients with OHS and coexistent severe OSA [11]. While beneficial, PAP therapy has been associated with weight gain in some studies [7]. Given that obesity is the primary driver of OHS pathophysiology, long-term management strategies must also prioritize weight reduction.

Bariatric surgery is a recognized and effective intervention for individuals with severe obesity and comorbidities like OSA [12]. We and others have shown that surgically induced weight loss can improve SDB and associated respiratory dysfunction [7,13]. Building on this, we hypothesize that a transdisciplinary approach combining airway and bariatric surgery (CABS) may provide a more comprehensive solution for OHS patients, whose condition involves overlapping mechanical, neuromuscular, and ventilatory impairments that may not be fully resolved by weight loss alone.

To our knowledge, this is the first study to apply a transdisciplinary CABS pathway specifically to the management of OHS. This prospective observational pilot had three objectives: (1) estimate the prevalence of OHS among patients with obesity and obstructive sleep apnea and identify associated risk factors; (2) derive and test clinical predictors of OHS; and (3) assess feasibility, establish proof-of-concept, and characterize early therapeutic responses to a single-session CABS intervention. These preliminary data are intended to evaluate safety and generate hypotheses about clinical benefit, thereby informing the design of larger, phenotype-driven trials.

## 2. Materials and Methods

### 2.1. Ethical Considerations

This study was conducted under the approval of the Institutional Review Board of the Chang Gung Medical Foundation (IRB No. 202201000A3, approved 8 August 2022) [13]. All participants provided written informed consent before any study-related procedures were initiated. The research adhered to internationally accepted ethical standards, including the principles outlined in the Declaration of Helsinki [14]. Reporting and methodological transparency followed established guidelines for observational research, particularly the STROCSS recommendations [15].

### 2.2. Study Design and Setting

The study was designed as a prospective cohort investigation over a two-year period, taking place at the Linkou Chang Gung Memorial Hospital Sleep Center, a tertiary referral facility specializing in complex SDB (1 December 2022–30 November 2024) [13]. The center’s high patient volume and comprehensive diagnostic resources allowed for consistent recruitment and structured follow-up.

OHS assessment relied on a multidimensional diagnostic approach incorporating anthropometric measurements, daytime gas-exchange evaluation, and sleep-related respiratory assessment. Participants were diagnosed with OHS if they exhibited obesity (BMI ≥ 30 kg/m^2^), persistent daytime hypercapnia (arterial PaCO_2_ ≥ 45 mmHg measured via standardized arterial blood gas sampling [ABG]) [7], and objective evidence of SDB. OSA was verified through overnight polysomnography. To ensure that hypercapnia originated from obesity-related ventilatory impairment rather than other medical conditions, each participant underwent pulmonary function testing and clinical evaluation to rule out neuromuscular respiratory disorders, chest wall restriction, or intrinsic lung disease. This diagnostic strategy aligns with current recommendations from professional respiratory societies, including the American Thoracic Society and European Respiratory Society, for evaluating chronic hypercapnic respiratory failure [7,16].

### 2.3. Inclusion and Exclusion Criteria

Participants were eligible if they were between 20 and 65 years of age and met two primary criteria: a BMI of at least 30 kg/m^2^ and an obstructive AHI of ≥5 events per hour. Individuals were excluded if they exhibited uncompensated hypercapnic respiratory failure (arterial pH < 7.30) [17], required invasive mechanical ventilation for acute respiratory compromise, or had underlying neuromuscular disorders, chest wall deformities, or significant pulmonary disease capable of causing hypoventilation independent of obesity. Additional exclusion criteria included unstable coronary artery disease and cognitive limitations that would interfere with informed consent or adherence to study procedures.

### 2.4. Interventions

**Figure 1:**
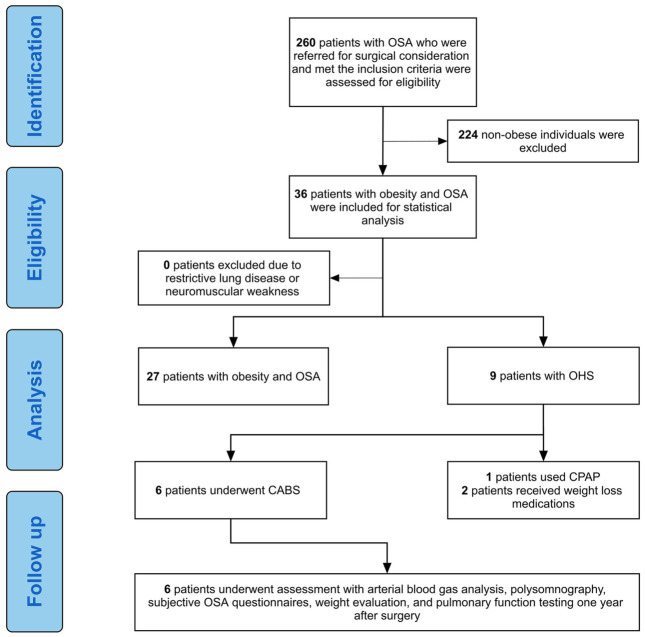
STROCSS flow diagram of participant recruitment and study procedures. This flow diagram illustrates the participant recruitment and study process, detailing initial patient assessment, BMI-based exclusions, and the final inclusion of 36 patients with obesity and OSA. It further outlines their stratification into OHS and non-OHS groups (based on arterial blood gas analysis), the treatment pathways for the OHS subgroup (CABS, CPAP, BWR), and the specific parameters assessed during follow-up for the CABS cohort. *Note.* BMI: body mass index; BWR: body weight reduction; CABS: combined airway and bariatric surgery; CPAP: continuous positive airway pressure; OHS: obesity hypoventilation syndrome; OSA: obstructive sleep apnea.

After receiving a confirmed diagnosis of OHS, patients met with the clinical team to review available treatment approaches. Management options included CPAP, structured weight-loss programs, and CABS. Treatment selection was individualized and determined through a shared decision-making discussion between clinicians and patients (Figure 1). Because participants chose their own treatment pathway, the study design was inherently non-randomized and therefore susceptible to selection bias. Subsequent analyses focused primarily on individuals who elected to undergo the CABS procedure.

#### 2.4.1. Trans-Disciplinary Intervention

Patients who elected the CABS approach underwent a coordinated operative plan under general anesthesia, during which bariatric surgery and multilevel airway reconstruction were completed in a single session. This collaborative model was designed to simultaneously address obesity-related ventilatory impairment and SDB. The bariatric portion of the operation was performed by a general surgeon, followed immediately by airway procedures carried out by an otorhinolaryngologist [13].

Laparoscopic sleeve gastrectomy (LSG) served as the principal bariatric technique [18]. Roux-en-Y gastric bypass was reserved for patients with substantial gastroesophageal reflux or poorly controlled diabetes mellitus [19]. For LSG, a 32-French bougie guided gastric calibration, and resection began approximately 4 cm above the pylorus, extending to within 1 cm of the angle of His. Preoperative endoscopy was used to exclude hiatal hernia. Staple lines were reinforced with seromuscular sutures. The operative steps for LSG have been detailed extensively in the prior surgical literature [20].

Airway surgery was individualized based on each patient’s anatomical features and pattern of upper-airway collapse. Surgical planning incorporated a comprehensive preoperative evaluation, including symptom assessment, awake anatomical examination (Friedman staging system) [21,22,23], lateral cephalometry [24], flexible nasopharyngoscopy [25], and drug-induced sleep endoscopy [26,27]. Only sites judged to contribute meaningfully to obstruction were addressed, resulting in tailored surgical combinations rather than a uniform procedure for all patients. Nasal surgery was performed for patients with clinically significant septal deviation or turbinate hypertrophy causing nasal blockage and mouth breathing. Palatal procedures were used when DISE demonstrated velopharyngeal collapse, whereas tongue-base surgery was reserved for individuals with posterior tongue obstruction. A partial epiglottectomy was undertaken in the single patient with epiglottic collapse identified on DISE.

**Figure 2:**
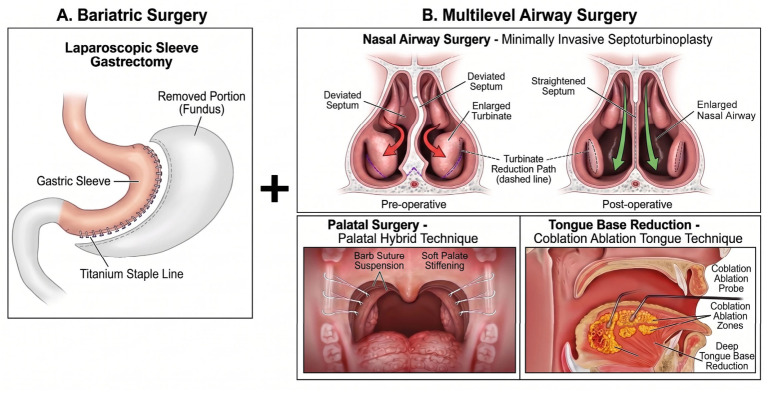
Surgical components of combined airway and bariatric surgery. This illustration depicts the integrated surgical approach used in the study, which combines bariatric surgery—primarily (**A**) laparoscopic sleeve gastrectomy—with (**B**) multilevel airway surgery. The airway procedures include nasal surgery (minimally invasive septoturbinoplasty), palatal surgery (palatal hybrid technique), and tongue base reduction (coblation ablation tongue technique) [13]. Red arrows denote sites of obstructed or disturbed airflow, whereas green arrows indicate restored and unobstructed nasal breathing following surgical intervention.

Multilevel airway reconstruction targeted obstruction at the nasal, palatal, and lingual levels. Nasal procedures followed a minimally invasive septoturbinoplasty approach, incorporating removal of deviated septal segments, drainage along the nasal floor, placement of trans-septal sutures to prevent hematoma formation, and controlled out-fracture of the inferior turbinates [28]. Palatal surgery used a hybrid technique [25] that combined mucosa-preserving tonsillectomy, reduction of supratonsillar fat, three-dimensional reconstruction of the tonsillar fossa, suspension of the soft palate using a raphe-based method, and anterior repositioning of the uvula. Lingual procedures were performed using coblation-based tongue reduction, either through lingual tonsillectomy with tongue-base contouring [29] or through multiple targeted coblation ablations directed at the intrinsic tongue musculature and adipose tissue of the tongue base (Figure 2). A partial epiglottectomy was performed in the patient who demonstrated epiglottic collapse on DISE [30].

#### 2.4.2. Postoperative Care

After surgery, patients were monitored in the recovery unit and began oral intake with clear liquids on the first postoperative day. Dietary progression was individualized, but most patients were able to transition to soft, easily digestible foods on the second day as their tolerance improved. Discharge was generally feasible by the fifth postoperative day, provided that patients maintained stable vital signs, had manageable pain, and showed no evidence of surgical or respiratory complications.

### 2.5. Outcome Measures

#### 2.5.1. Polysomnography

Overnight, attended level-I polysomnography was performed for all participants using the Nicolet UltraSom System (Nicolet, Madison, WI, USA). The AHI served as the principal metric of SDB. Apneas were defined as ≥90% reduction in airflow lasting at least 10 s, while hypopneas required >50% reduction in airflow accompanied by either a ≥3% drop in oxygen saturation or an arousal [31]. All studies were scored by a single sleep specialist who was blinded to clinical data.

#### 2.5.2. Pulmonary Function Testing

Spirometric assessment was conducted with a handheld spirometer following international standards. Measurements included forced vital capacity (FVC), forced expiratory volume in one second (FEV_1_), and the FEV_1_/FVC ratio [32].

#### 2.5.3. Gas Exchange

Daytime ABG sampling was performed with participants seated and breathing room air. To minimize variability, measurements were obtained after at least one hour of rest [33]. PaCO_2_ (mmHg), PaO_2_ (mmHg), and serum HCO_3_^−^ (mmol/L) were recorded.

#### 2.5.4. Epworth Sleepiness Scale

Subjective daytime sleepiness was evaluated using the Epworth Sleepiness Scale (ESS), an eight-item questionnaire with scores ranging from 0 to 24. Higher scores reflect greater levels of sleepiness [34].

### 2.6. Sample Size Calculation

The sample size was derived by examining the anticipated difference in the minimum SpO_2_ between individuals with OHS and those with non-OHS. Based on data from a previous study [5], which reported minimum SpO_2_ values of 64.2 ± 18.1% for OHS patients and 78.1 ± 10.2% for obesity and OSA patients, an effect size (Cohen’s d) of 0.95 was used for power calculations. To detect a difference in this magnitude with 80% statistical power and a two-sided α of 0.05, a Mann–Whitney *U* test required a minimum of 32 participants. Anticipating that roughly 10% of enrolled individuals might withdraw or be lost to follow-up, the target enrollment was increased to 36. All computations were performed using G*Power version 3.1.9.7 [35].

### 2.7. Statistical Analysis

Baseline demographic and clinical variables were summarized using descriptive methods. Continuous measurements were expressed as medians with interquartile ranges (IQRs), whereas categorical data were presented as counts and percentages. The proportion of participants meeting diagnostic criteria for OHS among those with both obesity and OSA was used to estimate OHS prevalence. Participants were subsequently stratified into OHS and non-OHS groups for comparative analyses.

Differences between groups were examined using the Mann–Whitney *U* test for non-normally distributed continuous variables and the Fisher exact test for categorical variables. To identify factors independently associated with OHS, a multivariable logistic regression model was constructed. Variables were entered using a forward selection strategy, and results were reported as odds ratios (ORs) with corresponding 95% confidence intervals (CIs).

Receiver operating characteristic (ROC) curves were generated to determine optimal thresholds for BMI, ESS scores, and the composite OHS prediction score. Diagnostic performance was quantified using the area under the curve (AUC). A confusion matrix was used to evaluate the classification behavior of the derived prediction score [36], including counts of true positives (TP), true negatives (TN), false positives (FP), and false negatives (FN). From these values, several performance indices were calculated:

Accuracy = (TP + TN)/(TP + TN + FP + FN)

Sensitivity (TP rate) = TP/(TP + FN)

Specificity (TN rate) = TN/(FP + TN)

F1 score = 2 × TP/(2 × TP + FP + FN)

For patients undergoing CABS, paired comparisons of pre- and postoperative measurements were performed using the Wilcoxon signed-rank test for paired samples. All statistical tests were two-sided, and significance was defined as *p* < 0.05. Statistical procedures were performed using IBM SPSS Statistics version for windows (Version 29.0; IBM Corp., Armonk, NY, USA).

### 2.8. Use of Artificial Intelligence and Assisted Technologies

During the preparation of this manuscript, artificial intelligence (AI) technologies were utilized specifically to assist with language refinement and the drafting of illustrative figures. Generative AI language models were employed to improve the readability, syntax, and structural clarity of the text. Additionally, AI-assisted graphic tools were utilized to generate the foundational visual layouts for the mechanistic diagrams outlining exploratory phenotypes. The authors rigorously reviewed, extensively edited, and independently verified all AI-generated text and visual elements against current literature. The authors maintain full responsibility for the originality, validity, and integrity of the final content, confirming that all scientific interpretations are solely the product of human intellect and that the manuscript complies fully with all publication ethics policies.

## 3. Results

### 3.1. Cohort Profile and Prevalence of OHS

Thirty-six adults with morbid obesity and OSA were included in the analysis (30 [83%] men and six [17%] women; median age, 45 years [IQR: 34–52]; median BMI, 35.0 kg/m^2^ [IQR: 31.3–38.9]; median AHI, 73.0 events/h [IQR: 37.2–99.5]). ABG analysis identified hypercapnia in nine participants, corresponding to a 25% prevalence of OHS within the cohort. All individuals with hypercapnia had no other identifiable causes of hypoventilation, as their FEV_1_/FVC ratios were greater than 70%. The OHS cohort was entirely male.

### 3.2. Comparison Between Participants with and Without OHS

The demographic profiles of participants with OHS were similar to those without OHS, with no meaningful differences in age or sex distribution (Table 1). Distinct physiologic differences, however, were evident. Individuals with OHS had a higher median BMI than their non-OHS counterparts (38.1 kg/m^2^ [IQR, 37.3–43.8] vs. 34.2 kg/m^2^ [IQR, 30.4–37.3]; *p* < 0.01) and lower minimum SpO_2_ (42% [IQR, 40–65] vs. 70% [IQR, 56–78]; *p* = 0.01) in the OHS group. Although the OHS group also exhibited higher scores on the ESS (14 vs. 12; *p* = 0.07) and greater AHI (93.8 events/h vs. 69.7 events/h; *p* = 0.08), the elevations noted in the OHS group did not attain statistical significance.

### 3.3. Predictors of OHS and Predictive Model Performance

#### 3.3.1. Univariate Analysis

In the initial logistic regression screening, two variables demonstrated meaningful associations with the presence of OHS. Higher BMI was linked to increased odds of OHS (OR, 1.31; 95% CI, 1.07–1.61; *p* = 0.01), and lower minimum nocturnal SpO_2_ also correlated with OHS (OR, 0.92; 95% CI, 0.86–0.98; *p* = 0.01). These models accounted for a moderate proportion of variance (Nagelkerke *R*^2^ = 0.35 and 0.32, respectively). No significant associations were observed for sex, age, ESS, or AHI in univariate models.

#### 3.3.2. Multivariable Analysis

A forward-selection multivariable logistic regression model identified BMI (OR, 1.69; 95% CI, 1.08–2.62; *p* = 0.02) and ESS (OR, 1.90; 95% CI, 1.04–3.46; *p* = 0.04) as independent predictors of OHS after adjustment for sex. The model explained a substantial proportion of variance (Nagelkerke *R*^2^ = 0.77). The extremely large weight assigned to female sex in the model reflects the fact that none of the OHS cases in this small cohort were female.

#### 3.3.3. OHS Prediction Score and Performance

From this model, a preliminary OHS prediction score was derived:

$$OHS\;prediction\;score=\frac{100}{1+e^{-(-28.09-20.54\times female\;sex+0.52\times BMI+0.64\times ESS)}}$$

**Figure 3:**
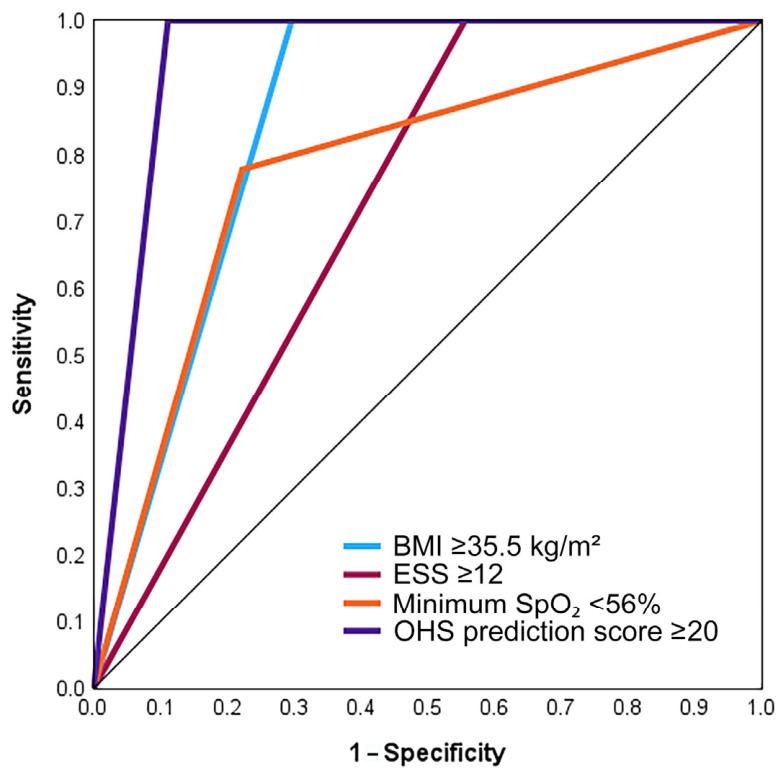
Receiver operating characteristic (ROC) curves for predicting obesity hypoventilation syndrome (OHS). The graph depicts the diagnostic performance of various clinical predictors. The *x*-axis represents 1 − specificity, and the *y*-axis represents sensitivity. Among the predictors, an OHS prediction score ≥ 20 (purple line) demonstrated the largest area under the curve (AUC), indicating superior diagnostic accuracy compared to BMI ≥35.5 kg/m^2^ (light blue line), Epworth Sleepiness Scale (ESS) ≥ 12 (maroon line), and minimum peripheral capillary oxygen saturation (SpO_2_) < 56% (orange line).

Using Youden’s index [37], a threshold of ≥20 was identified as the optimal cut-point. The score demonstrated strong discriminative ability, with an AUC of 0.94 (95% CI, 0.87–1.02; *p* < 0.01) (Figure 3). At this cut-point, the model achieved an accuracy of 92%, sensitivity of 100%, specificity of 89%, and an F1 score of 86%.

#### 3.3.4. Individual Predictor Performance

Further ROC analyses highlighted the predictive utility of individual variables. A BMI threshold of ≥35.5 kg/m^2^ demonstrated strong performance (AUC, 0.85; 95% CI, 0.73–0.97; *p* < 0.01), with an accuracy of 78%, sensitivity of 100%, specificity of 70%, and an F1 score of 69%. The minimum recorded SpO_2_ value of <56% also emerged as a significant predictor (AUC, 0.78; 95% CI 0.59–0.96; *p* = 0.01), achieving an accuracy of 80%, sensitivity of 88%, specificity of 78%, and an F1 score of 67%. Similarly, an ESS score of ≥12 exhibited predictive utility (AUC, 0.72; 95% CI 0.56–0.89; *p* = 0.01), though with a lower overall accuracy of 58%, sensitivity of 100%, specificity of 44%, and an F1 score of 55%.

### 3.4. Feasibility and Early Outcomes of CABS

Six men with confirmed OHS elected to undergo CABS after shared decision-making (Table 2). Their median age was 46 years (IQR 33–55), median BMI 40.1 kg/m^2^ (IQR 37.6–45.5), and median AHI 98.1 events/h (IQR 70.4–113.4), reflecting severe obesity and marked SDB. Airway phenotypes varied considerably, with differences in Friedman stage and DISE-identified collapse at the velum, oropharynx, tongue base, and epiglottis. Airway procedures were selected according to these findings: nasal and palatal surgery were common, tongue-base surgery was used only when posterior tongue obstruction was evident, and epiglottic surgery was performed solely in the patient with epiglottic collapse. One patient (Patient 6) required two lingual techniques because of pronounced lingual tonsillar hypertrophy. All individuals underwent laparoscopic sleeve gastrectomy.

The combined procedure was technically feasible in all cases. No perioperative or major postoperative complications occurred, indicating that the coordinated surgical approach was technically feasible and well-tolerated.

At one-year follow-up, statistically significant improvements were observed across all primary clinical parameters (all *p* = 0.03) except for serum HCO_3_^−^ and FEV_1_/FVC ratio (Table 3).

#### 3.4.1. Resolution of Hypercapnia

**Figure 4:**
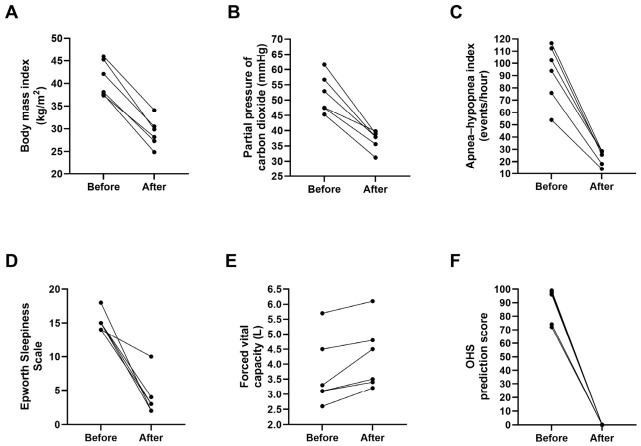
Postoperative changes in (**A**) body mass index, (**B**) partial pressure of carbon dioxide, (**C**) apnea–hypopnea index, (**D**) Epworth Sleepiness Scale, (**E**) forced vital capacity, and (**F**) obesity hypoventilation syndrome (OHS) prediction score.

All six treated patients (100%) achieved normocapnia. Median PaCO_2_ declined from 50.2 mmHg to 37.9 mmHg (*p* = 0.03) (Figure 4A).

#### 3.4.2. Weight Loss

Median BMI decreased from 40.1 kg/m^2^ to 29.1 kg/m^2^ (*p* = 0.03) (Figure 4B).

#### 3.4.3. Sleep Parameters

Median AHI decreased by 73% (from 98.1 to 26.8 events/h; *p* = 0.03) (Figure 4C), and median ESS scores normalized (from 15 to 3; *p* = 0.03) (Figure 4D).

#### 3.4.4. Pulmonary Mechanics

Median FVC increased significantly from 3.2 L to 4.0 L (*p* = 0.03) (Figure 4E), suggesting improved chest wall compliance.

#### 3.4.5. OHS Prediction Score

Median OHS prediction score significantly reduced from 97 to 0 after CABS (Figure 4F).

While OHS was resolved in all surgical patients, residual OSA persisted; two patients (33%) had residual obesity and OSA, while four (67%) had residual OSA alone.

## 4. Discussion

In this prospective pilot study, we examined the burden of OHS among individuals with morbid obesity and OSA, and evaluated early signals of benefit from a CABS strategy. Several observations emerged. First, OHS was not uncommon in this cohort, affecting one in four participants. Second, higher BMI and greater daytime sleepiness were the clinical features most closely associated with OHS. Third, patients who underwent the transdisciplinary CABS procedure demonstrated improvements across selected respiratory and metabolic domains.

Although OHS can occur independently of sleep-disordered breathing, the two conditions frequently overlap. Prior reports indicate that the majority of patients with OHS also have OSA, with estimates approaching 90% in some clinical samples [9]. Much of the existing literature is derived from sleep-center populations, where the prevalence of OHS among individuals with OSA has ranged widely—from single-digit percentages to nearly half of all patients—reflecting differences in referral patterns, obesity severity, and diagnostic criteria [39,40]. East Asian cohorts add further nuance; craniofacial structure and airway morphology may predispose individuals to OSA at lower BMI thresholds [41], and studies from Japan have reported OHS prevalence rates of 14% and 38% among patients with OSA [42,43]. In our cohort of adults with morbid obesity and OSA, one quarter met diagnostic criteria for OHS, and all had severe OSA. This proportion aligns with the higher end of previously reported ranges and reinforces the importance of considering OHS when evaluating patients with obesity and marked SDB. The absence of female cases in our sample likely reflects the small cohort size rather than sex-specific biological protection, but it illustrates how OHS may be underrecognized in demographically imbalanced clinical settings (Table 4).

**Figure 5:**
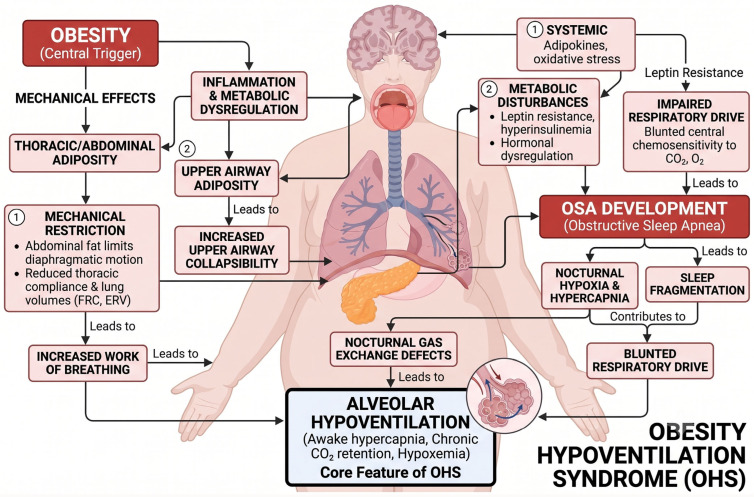
Detailed mechanistic pathways linking obesity, obesity and obstructive sleep apnea (OSA), and hypoventilation to obesity hypoventilation syndrome (OHS). ERV: expiratory reserve volume; FRC: functional residual capacity.

In our cohort, individuals with OHS demonstrated a distinct clinical profile, characterized by greater adiposity and more pronounced daytime sleepiness compared with those who had OSA alone [44]. When examined in multivariable models, BMI remained the strongest independent correlate of OHS, reinforcing the central role of excess body mass in the development of chronic ventilatory failure [39,45]. The pathophysiology of OHS reflects a convergence of several processes rather than a single abnormality (Figure 5).

One major contributor is the mechanical load imposed by thoracic and abdominal adiposity, which limits diaphragmatic excursion, reduces chest wall compliance, and lowers lung volumes—particularly functional residual capacity and expiratory reserve volume—ultimately increasing the work of breathing [46,47]. Metabolic and inflammatory disturbances associated with obesity further compound these mechanical constraints. Leptin resistance, oxidative stress, and neurohormonal dysregulation blunt central chemosensitivity to CO_2_ and O_2_, diminishing ventilatory drive [48,49]. These systemic alterations also promote soft-tissue deposition around the upper airway, increasing collapsibility and facilitating the development of OSA. Recurrent nocturnal hypoxemia, hypercapnia, and sleep fragmentation then further depress ventilatory responsiveness and destabilize gas exchange, creating a physiologic environment conducive to sustained alveolar hypoventilation [50,51].

The interaction of these mechanical, metabolic, and sleep-related disturbances places individuals with obesity and OSA at heightened risk for OHS. Those who develop both disorders often experience more severe downstream consequences, including pulmonary hypertension, cardiovascular morbidity, and increased mortality [44]. Although the dominant mechanisms involve impaired ventilatory drive and mechanical restriction, additional minor factors—such as chronic low-grade inflammation, respiratory muscle inefficiency, reduced lung compliance, metabolic dysfunction, and attenuated chemosensitivity—may further exacerbate ventilatory instability.

Although ABG analysis remains the definitive method for confirming OHS [11], its invasiveness limits its practicality as a first-line screening tool. Identifying simple, clinically accessible markers may help clinicians recognize high-risk individuals earlier. In this cohort, BMI ≥ 35.5 kg/m^2^ and ESS ≥ 12 served as useful thresholds, and a composite score incorporating BMI, ESS, and sex demonstrated strong discriminative ability. These findings suggest that straightforward clinical indicators may help guide decisions about when ABG testing is warranted, though validation in larger and more heterogeneous populations is needed before such an approach can be broadly implemented.

Several other noninvasive measures have been explored as potential screening aids. Serum bicarbonate and nocturnal oxygen saturation indices have shown moderate predictive value in prior studies [11,52,53], and incremental increases in BMI have consistently been associated with higher OHS risk [54]. These observations highlight the importance of obesity-related metrics in clinical assessment, even though none of these tools alone can replace ABG confirmation.

Transcutaneous carbon dioxide monitoring (tcPCO_2_) has gained attention as an adjunctive method for detecting hypercapnia, particularly in bariatric candidates at elevated risk for ventilatory impairment [44,55]. Because tcPCO_2_ correlates reasonably well with arterial PaCO_2_ and avoids repeated arterial puncture [55], it may be useful for preoperative screening and longitudinal follow-up. Nonetheless, ABG analysis remains the diagnostic gold standard, and tcPCO_2_ should be regarded as a complementary modality rather than a substitute.

PAP therapy continues to serve as the foundational treatment for OHS, especially when severe OSA is present [7]. By stabilizing the upper airway and improving nocturnal ventilation, PAP reduces obstructive events, improves oxygenation, and lowers carbon dioxide retention (Table 5) [56]. These physiologic benefits provide essential stabilization for patients with OHS. However, PAP does not directly address the underlying mechanical and metabolic consequences of obesity, and long-term adherence can be challenging. For these reasons, PAP is best integrated into a broader multidisciplinary strategy that may include lifestyle modification, glucagon-like peptide-1 receptor agonists, bariatric surgery, and targeted airway procedures based on individual anatomy and shared decision-making [57,58].

Screening for OHS is particularly important in bariatric surgery candidates, who often present with overlapping obesity, severe OSA, and ventilatory impairment [59]. Unrecognized OHS has been associated with increased perioperative risk, including difficult airway management, postoperative respiratory failure, prolonged ventilation, ICU admission, and cardiopulmonary complications [60]. Early identification allows clinicians to optimize perioperative care through tailored anesthetic planning, appropriate respiratory support, enhanced postoperative monitoring, and timely initiation of PAP therapy. It also facilitates individualized long-term treatment planning, including medical weight reduction, surgical intervention, and management of persistent upper-airway obstruction [61]. Our findings suggest that simple clinical indicators—such as BMI, ESS, and oxygen saturation—may help identify bariatric candidates who warrant confirmatory ABG testing before surgery.

Bariatric surgery is well established as an effective intervention for substantial weight reduction and improvement in ventilatory mechanics [62]. However, weight loss alone does not reliably eliminate upper-airway obstruction, and residual OSA is common even after successful metabolic surgery [63,64]. Recognizing that OHS arises from intertwined mechanical, metabolic, and airway-related factors, we examined a CABS strategy as a potential means of addressing these overlapping mechanisms. Airway surgery, when applied to appropriately selected patients, has been shown to improve both symptoms and objective respiratory parameters in OSA. In our previous comparative work, CABS produced greater reductions in AHI than bariatric surgery alone (65.7 vs. 31.5 events/h; *p* = 0.04) and achieved a higher overall surgical success rate (90% vs. 80%) [63].

In the present cohort, patients undergoing CABS demonstrated improvements across several domains, including AHI, BMI, PaCO_2_, and FVC, with all individuals achieving normocapnia at one year. These findings appear favorable relative to historical outcomes following bariatric surgery alone [63]. Even so, the absence of a bariatric-only control group prevents us from determining how much of the observed improvement can be attributed specifically to airway surgery. For this reason, the present study should be regarded as an early feasibility assessment rather than evidence of incremental efficacy. Larger prospective comparative studies are needed to clarify whether CABS provides additive benefit in OHS management.

Given the multifactorial nature of OHS, optimal care requires a coordinated strategy that addresses both obesity and SDB. CPAP remains the primary therapy for stabilizing nocturnal ventilation and correcting sleep-related hypoventilation, particularly in patients with severe OSA [65]. More recently, glucagon-like peptide-1 receptor agonists have emerged as a potent nonsurgical option for weight reduction and improvement of obesity-related metabolic dysfunction, offering an alternative pathway for selected patients [66]. Bariatric surgery continues to provide the most durable metabolic and ventilatory benefits, while airway surgery targets persistent upper-airway obstruction that may not fully resolve with weight loss alone.

Rather than competing approaches, these interventions should be viewed as complementary components of an integrated therapeutic framework. Treatment selection should be individualized, taking into account each patient’s anatomical features, metabolic profile, severity of SDB, comorbidities, and personal preferences. A multidisciplinary, phenotype-driven strategy—supported by shared decision-making—offers the greatest potential for achieving meaningful and sustained clinical improvement.

This study was designed to assess feasibility and early outcomes, not to define ideal indications for CABS. Future work should shift toward identifying which OHS phenotypes are most likely to benefit from combined surgery. Such phenotype-guided selection may integrate obesity severity, airway collapse patterns, OSA burden, daytime hypercapnia, ventilatory impairment, metabolic dysfunction, and other physiological endotypes. A precision-medicine approach may help match patients to the most appropriate interventions, optimize outcomes, and avoid unnecessary procedures in those unlikely to benefit from combined surgery.

## 5. Limitations

This study was designed as a preliminary feasibility evaluation rather than a comparative efficacy trial; therefore, the findings must be interpreted in the context of several key constraints.

First, the nonrandomized, single-center pilot design inherently restricts statistical power. With only thirty-six morbid obesity and OSA patients recruited and six OHS patients undergoing CABS, the small sample size limits the precision of our effect estimates and elevates the risk of selection bias.

Second, the study design lacks a control arm of patients undergoing bariatric surgery alone. Because CABS concurrently modifies the airway and facilitates weight loss, we cannot isolate the independent therapeutic contributions of the airway interventions from those achieved through systemic weight reduction.

Third, while our preoperative evaluations were thorough—incorporating awake anatomical assessments, cephalometry, and drug-induced sleep endoscopy—surgical decisions were not dictated by a prospectively validated, standardized algorithm. The heavy reliance on multilevel airway reconstruction in this early institutional cohort precludes us from evaluating the distinct efficacy of individual surgical components, which limits the translation of these findings into precision, phenotype-driven practice.

Fourth, the demographic profile of the cohort restricts external validity. Participants were recruited exclusively from a tertiary referral sleep center, and the OHS subgroup skewed heavily male, meaning these results may not accurately reflect broader community or typical bariatric populations.

Finally, the one-year follow-up restricts our outcome data to the short term. This timeframe is insufficient to establish surgical durability, identify late-onset complications, or track the long-term trajectory of residual OSA. Crucial patient-centered endpoints, including longitudinal cardiometabolic morbidity and health-related quality of life, were not assessed.

Given these constraints, CABS should strictly remain an investigational approach rather than being adopted as standard clinical practice. These preliminary data highlight the necessity for rigorous, multicenter prospective trials. Future investigations must incorporate bariatric-only control arms and standardized, phenotype-based selection protocols to accurately define which patient subgroups genuinely stand to benefit from adjunctive airway surgery.

## 6. Conclusions

In this prospective observational study, CABS was technically feasible and associated with improvements in weight, SDB, gas exchange, and pulmonary function in selected patients with OHS. One quarter of individuals with morbid obesity and OSA met criteria for OHS, with higher BMI and greater daytime sleepiness emerging as key clinical features. An exploratory prediction score incorporating BMI, ESS, and sex showed good discriminative potential.

Among patients who elected CABS, ventilatory and metabolic measures improved over one year, including normalization of PaCO_2_. These early results suggest that an integrated surgical approach may offer therapeutic value for appropriately selected patients. Larger, comparative studies will be needed to determine its broader applicability and to clarify whether CABS provides benefit beyond bariatric surgery alone.

## Figures and Tables

**Table 1:** Differences in clinical measures between the obesity hypoventilation syndrome (OHS) and non-OHS groups.

Parameter	OHS	Non-OHS	*p*-Value
No	9	27	
Sex, No. (female/male)	0/9	6/21	0.12
Age, years	42 (33–50)	45 (35–52)	0.49
BMI, kg/m^2^	38.1 (37.3–43.8)	34.2 (30.4–37.3)	**<0.01**
ESS, score	14 (14–15)	12 (8–15)	0.07
AHI, events/h	93.8 (64.9–116.2)	69.7 (35.4–90.4)	0.08
Minimum SpO_2_, %	42 (40–65)	70 (56–78)	**0.01**
PaCO_2_, mmHg	50.1 (47.5–54.8)	38.8 (36.5–42.1)	**<0.01**

Data are presented as median (interquartile range) unless otherwise indicated. Significant values are depicted in bold. AHI: apnea–hypopnea index; BMI: body mass index; ESS: Epworth Sleepiness Scale; PaCO_2_: arterial partial pressure of carbon dioxide; SpO_2_: peripheral capillary oxygen saturation.

**Table 2:** Preoperative characteristics, airway phenotypes, and individualized airway procedures in patients undergoing combined airway and bariatric surgery (*n* = 6).

Patient	Age	BMI	Friedman Staging System [22]			DISE [26]				AHI	Airway Surgery				Bariatric Surgery
	(Year)	(kg/m^2^)	TS	TP	Stage	V	O	T	E	(Events/h)	NS	PS	LS	ES	
1	59	37.4	1/1	III	III	2	1	2	0	53.9	✓	✓	✓ ^1^	—	LSG
2	45	45.3	1/1	III	IV	2	2	1	0	116.6	✓	✓	—	—	LSG
3	33	38.1	1/1	IIb	II	2	1	2	0	93.8	—	✓	✓ ^1^	—	LSG
4	32	37.6	2/2	III	III	2	2	1	2	75.9	✓	✓	—	✓	LSG
5	53	46.0	2/3	III	IV	2	1	1	0	102.4	✓	✓	—	—	LSG
6	46	42.1	2/2	IIb	IV	2	2	2	0	112.3	✓	✓	✓ ^2^	—	LSG

^1^ Lingual surgery performed using single coblation endoscopic lingual lightening [29]. ^2^ Lingual surgery performed using combined coblation endoscopic lingual lightening and coblation tongue-base ablation [38]. AHI: apnea–hypopnea index; BMI: body mass index; E: Epiglottitis; ES: epiglottic surgery; LS: lingual surgery; LSG: laparoscopic sleeve gastrectomy; NS: nasal surgery; O: oropharynx; PS: palatal surgery; T: tongue base; TP: tongue position; TS: tongue size; V: velum; ✓: yes.

**Table 3:** Perioperative changes in clinical parameters among individuals with obesity hypoventilation syndrome undergoing combined airway and bariatric surgery (*n* = 6).

Parameter	Preoperative	Postoperative	*p*-Value
VAS for snoring (0–10)	8 (7–10)	2.0 (0–3)	**0.03**
ESS score (0–24)	15 (14–16)	3 (2–6)	**0.03**
BMI, kg/m^2^	40.1 (37.4–46.0)	29.1 (24.9–34.1)	**0.03**
Body weight, kg	122.7 (104.5–134.0)	84.5 (76.0–100.0)	**0.03**
AHI, events/h	98.1 (54.0–117.0)	26.8 (14.0–28.6)	**0.03**
Minimum SpO_2_, %	40.0 (39.0–74.0)	78.0 (74.0–83.0)	**0.03**
PaCO_2_, mmHg	50.2 (45.3–61.6)	37.9 (31.2–39.8)	**0.03**
PaO_2_, mmHg	79.2 (62.0–80.9)	93.9 (80.5–108.0)	**0.03**
Serum HCO_3_^−^, mmol/L	26.5 (22.6–29.4)	24.8 (21.7–26.6)	0.35
FVC, L	3.2 (2.6–5.7)	4.0 (3.2–6.1)	**0.03**
FEV_1_, L	3.0 (2.3–4.9)	3.6 (2.9–5.1)	**0.03**
FEV_1_/FVC ratio, %	0.80 (0.70–0.90)	0.90 (0.80–0.90)	0.46
OHS prediction score	97 (74–99)	0 (0–0)	**0.03**

Data are presented as median (interquartile range) unless otherwise indicated. Significant values are depicted in bold. AHI: apnea–hypopnea index; BMI: body mass index; ESS: Epworth Sleepiness Scale; FEV_1_: forced expiratory volume in 1 s; FVC: forced vital capacity; HCO_3_^−^: serum bicarbonate; PaCO_2_: arterial partial pressure of carbon dioxide; PaO_2_: arterial partial pressure of oxygen; SpO_2_: peripheral oxygen saturation; VAS: visual analogue scale.

**Table 4:** Suggested clinical approach for identifying and confirming obesity hypoventilation syndrome (OHS).

Initial Screening	Physiologic Confirmation	Final Diagnosis Determination
Obesity (BMI ≥ 30 kg/m^2^) + SDB	Daytime ABG Analysis	OHS confirmed after excluding alternative causes of chronic hypoventilation
BMI ≥ 35.5 kg/m^2^	PaCO_2_ ≥ 45 mmHg	Neuromuscular disorders ruled out
ESS ≥ 12	—	Chest wall abnormalities excluded
Minimum nocturnal SpO_2_ < 56%	—	Pulmonary diseases contributing to hypoventilation excluded
OHS prediction score ≥ 20	—	—

ABG: arterial blood gas; BMI: body mass index; ESS: Epworth Sleepiness Scale; PaCO_2_: arterial partial pressure of carbon dioxide; SDB: sleep-disordered breathing; SpO_2_: peripheral oxygen saturation.

**Table 5:** Potential mechanisms of improvement with positive airway pressure therapy in obesity hypoventilation syndrome.

Therapeutic Effect	Physiologic Mechanism	Clinical Implication
Upper-airway stabilization	Splints the pharyngeal airway, reducing collapsibility and obstructive events	Improves OSA severity and sleep continuity
Enhanced nocturnal ventilation	Supports alveolar ventilation and reduces hypoventilation	Lowers nocturnal and daytime PaCO_2_
Improved oxygenation	Mitigates intermittent and sustained hypoxemia	Enhances gas exchange and tissue oxygen delivery
Reduced work of breathing	Decreases inspiratory load and respiratory muscle effort	Improves ventilatory efficiency
Lower sympathetic activation	Reduces arousal burden and hypoxemia-related stress	May lessen cardiovascular strain
Better sleep quality	Decreases apneas, hypopneas, and sleep fragmentation	Improves daytime alertness and quality of life
Cardiopulmonary unloading	Reduces hypoxemia- and hypercapnia-related pulmonary vascular stress	May decrease risk of pulmonary hypertension
Bridge to weight-loss therapy	Stabilizes ventilation during medical or surgical weight-loss interventions	Supports safer integrated long-term management

OSA: obstructive sleep apnea; PaCO_2_: arterial partial pressure of carbon dioxide.

## Data Availability

The datasets generated and/or analyzed during the current study are available from the corresponding author upon reasonable request.

## Abbreviations

The following abbreviations are used in this manuscript:

ABG	Arterial blood gas
AHI	Apnea-hypopnea index
AI	Artificial intelligence
AUC	Area under the curve
BMI	Body mass index
BWR	Body weight reduction
CABS	Combined airway and bariatric surgery
CIs	Confidence intervals
CPAP	Continuous positive airway pressure
ESS	Epworth Sleepiness Scale
FEV_1_	Forced expiratory volume in 1 s
FN	False negative
FP	False positive
FVC	Forced vital capacity
HCO_3_^−^	Serum bicarbonate
LSG	Laparoscopic sleeve gastrectomy
OHS	Obesity hypoventilation syndrome
ORs	Odds ratios
OSA	Obstructive sleep apnea
PaCO_2_	Arterial partial pressure of carbon dioxide
PaO_2_	Arterial partial pressure of oxygen
PAP	Positive airway pressure
PFT	Pulmonary function test
ROC	Receiver operating characteristic
SDB	Sleep-disordered breathing
SpO_2_	Peripheral oxygen saturation
STROCSS	Strengthening the Reporting of Observational Studies in Epidemiology
tcPCO_2_	Transcutaneous carbon dioxide
TN	True negative
TP	True positive
VAS	Visual analogue scale
